# *N* ^2^-(7-Bromo-9-butyl-9*H*-carbazol-2-yl)-9,9-diethyl-*N* ^2^,*N* ^7^,*N* ^7^-triphenyl-9*H*-fluorene-2,7-diamine

**DOI:** 10.1107/S160053681200791X

**Published:** 2012-02-29

**Authors:** Abhishek Baheti, K. R. Justin Thomas, Seik Weng Ng, Edward R. T. Tiekink

**Affiliations:** a Organic Materials Laboratory, Department of Chemistry, Indian Institute of Technology Roorkee, Roorkee 247 667, India; b Department of Chemistry, University of Malaya, 50603 Kuala Lumpur, Malaysia; c Chemistry Department, Faculty of Science, King Abdulaziz University, PO Box 80203 Jeddah, Saudi Arabia

## Abstract

In the title mol­ecule, C_51_H_46_BrN_3_, the central fluorene residue is planar (r.m.s. deviation = 0.0203 Å), as is the carbazole system (r.m.s. deviation = 0.0154 Å), and these groups are almost orthogonal [dihedral angle = 79.72 (3)°]. The three-dimensional architecture is consolidated by C—H⋯π inter­actions. The butyl substituent is disordered with two sites resolved for the terminal propyl atoms; the major component had a site-occupancy factor of 0.686 (3).

## Related literature

For the use of carbazole and fluorene derivatives as hole-transporting and emitting materials in organic light-emitting diodes and as sensitizers in dye-sensitized solar cells, see: Thomas *et al.* (2001 ▶, 2004 ▶); Baheti *et al.* (2009 ▶, 2011 ▶). For related structures, see: Low *et al.* (2005 ▶); Chen *et al.* (2009 ▶); Gagnon & Laliberté (2008 ▶).
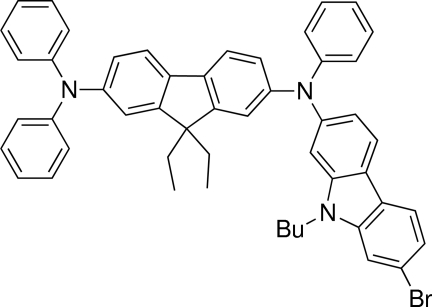


## Experimental

### 

#### Crystal data

C_51_H_46_BrN_3_
*M* *_r_* = 780.82
Monoclinic, 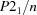

*a* = 8.6585 (2) Å
*b* = 10.6744 (2) Å
*c* = 43.6607 (6) Å
β = 92.114 (2)°
*V* = 4032.56 (13) Å^3^
*Z* = 4
Cu *K*α radiation
μ = 1.68 mm^−1^
*T* = 100 K
0.22 × 0.18 × 0.14 mm

#### Data collection

Agilent SuperNova Dual diffractometer with an Atlas detector
Absorption correction: multi-scan (*CrysAlis PRO*; Agilent, 2010 ▶) *T*_min_ = 0.836, *T*_max_ = 1.000
17423 measured reflections
8220 independent reflections
7219 reflections with *I* > 2σ(*I*)
*R*_int_ = 0.025

#### Refinement

*R*[*F* ^2^ > 2σ(*F* ^2^)] = 0.044
*wR*(*F* ^2^) = 0.112
*S* = 1.05
8220 reflections
506 parameters
28 restraints
H-atom parameters constrained
Δρ_max_ = 0.39 e Å^−3^
Δρ_min_ = −0.66 e Å^−3^

### 

Data collection: *CrysAlis PRO* (Agilent, 2010 ▶); cell refinement: *CrysAlis PRO*; data reduction: *CrysAlis PRO*; program(s) used to solve structure: *SHELXS97* (Sheldrick, 2008 ▶); program(s) used to refine structure: *SHELXL97* (Sheldrick, 2008 ▶); molecular graphics: *ORTEP-3* (Farrugia, 1997 ▶) and *DIAMOND* (Brandenburg, 2006 ▶); software used to prepare material for publication: *publCIF* (Westrip, 2010 ▶).

## Supplementary Material

Crystal structure: contains datablock(s) global, I. DOI: 10.1107/S160053681200791X/bt5825sup1.cif

Structure factors: contains datablock(s) I. DOI: 10.1107/S160053681200791X/bt5825Isup2.hkl

Supplementary material file. DOI: 10.1107/S160053681200791X/bt5825Isup3.cml

Additional supplementary materials:  crystallographic information; 3D view; checkCIF report

## Figures and Tables

**Table 1:** Hydrogen-bond geometry (Å, °) *Cg*1–*Cg*3 are the centroids of the C13–C18, C7–C12 and C36⋯C41 rings, respectively.

*D*—H⋯*A*	*D*—H	H⋯*A*	*D*⋯*A*	*D*—H⋯*A*
C10—H10⋯*Cg*1^i^	0.95	2.67	3.444 (3)	139
C20—H20⋯*Cg*2^ii^	0.95	2.85	3.579 (2)	135
C44—H44⋯*Cg*3^iii^	0.95	2.99	3.836 (3)	149

Symmetry codes: (i) 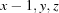; (ii) 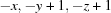; (iii) 
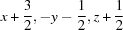
.

**Fig. 1.**
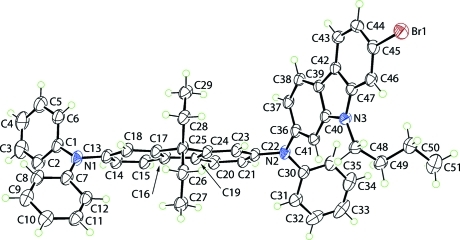
The molecular structure of (I) showing the atom-labelling scheme and displacement ellipsoids at the 50% probability level. Only the major component of the disordered n-butyl group is shown for reasons of clarity.

**Fig. 2.**
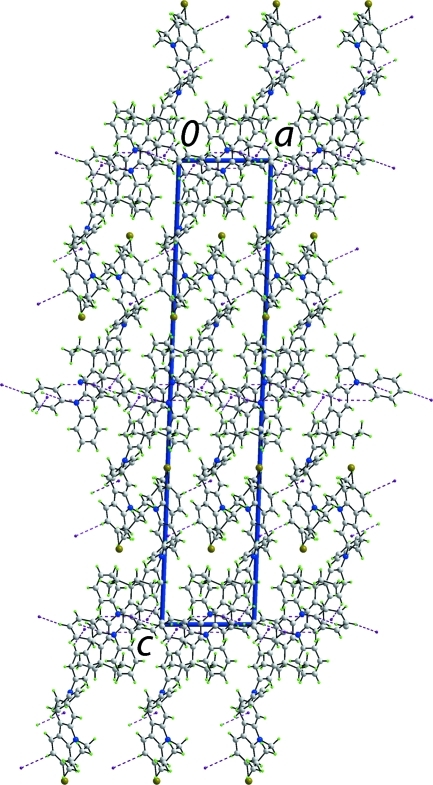
A view in projection down the b axis of the unit-cell contents of (I). The C—H···π interactions are shown as purple dashed lines.

## supplementary crystallographic information

### Comment

Carbazole (Thomas *et al.*, 2001; Thomas *et al.*, 2004) and fluorene (Baheti *et al.*, 2009; Baheti *et al.*, 2011) derivatives have been widely used as hole-transporting and emitting materials in organic light-emitting diodes and as sensitizers in dye-sensitized solar cells. The title compound, *N*^2^-(7-bromo-9-butyl-9*H*-carbazol-2-yl)-9,9-diethyl-*N*^2^,*N*^7^,*N*^7^-triphenyl-9*H*-fluorene-2,7-diamine (I), was synthesized as an intermediate in the synthetic sequence for the development of donor-acceptor compounds suitable for application as sensitizers in dye-sensitized solar cells.. Herein, the crystal structure determination of (I) is described. Related structures are known, *i.e*. 9,9-diethyl-*N*^2^,*N*^2^,*N*^7^,*N*^7^-tetraphenyl-9*H*-fluorene-2,7-diamine (Low *et al.*, 2005), 9-butyl-9*H*-carbazole (Chen *et al.*, 2009) and 2,7-dibromo-9-octyl-9*H*-carbazole (Gagnon & Laliberté, 2008).

In (I), Fig. 1, the 13 non-hydrogen atoms of the central fluorene residue are co-planar (r.m.s. deviation = 0.0203 Å). Similarly, the 13 non-hydrogen atoms of the carbazole system forms a plane (r.m.s. deviation = 0.0154 Å). The dihedral angle between the fluorene and carbazole fused ring systems is 79.72 (3)°, indicating an almost orthogonal relationship. The fluorene system forms dihedral angles of 88.22 (6) and 58.51 (7)° with the C1–C6 and C7–C12 N1-amine-phenyl rings, respectively; the dihedral angle between these phenyl rings is 64.45 (8) °. At the N2-amine side of the molecule, the dihedral angles formed between the fluorene and carbazole fused ring systems with the N2-bound phenyl ring are 62.96 (6) and 71.53 (6)°, respectively. With respect to the five-membered ring in the fluorene residue, the ethyl substituents are virtually perpendicular as seen in the values of the C17–C25–C26–C27 and C17–C25–C28–C29 torsion angles of 54.4 (3) and -60.6 (3)°, respectively. Finally, the major component of the disordered *n*-butyl chain adopts an extended *trans* conformation with the C48–C49–C50–C51 torsion angle being 173.2 (3)°; the equivalent value for the minor component is 179.8 (8)°.

Molecules are consolidated in the three-dimensional architecture by C—H···π interactions, Fig. 2 and Table 1.

### Experimental

A mixture of 9,9-diethyl-*N*^2^,*N*^2^,*N*^7^-triphenyl-9*H*-fluorene-2,7-diamine, 2,7-dibromo-9-butyl-9*H*-carbazole, bis(diphenylphosphino)ferrocene, sodium *tert*-butoxide, and toluene (20 ml) was heated at 353 K for 36 h. The resultant solution was poured into water and extracted with dichloromethane. After drying over anhydrous sodium sulfate the combined dichloromethane extract was evaporated to yield a crude product. It was purified by column chromatography on silica gel using 4:1 mixture of hexanes and dichloromethane. White solid. Yield 66%; *M*.pt. 413–415 K. Re-crystallization was by slow evaporation of a solution of (I) from a 1:1 mixture of hexanes and dichloromethane. ^1^H NMR (500 MHz, CDCl_3_) δ 0.39 (t, J = 7.5 Hz, 6 H), 0.85–0.88 (m, 3 H), 1.29 (dd, J = 15.5 Hz, 7.5 Hz, 2 H), 1.71 (t, J = 7.5 Hz, 2 H), 1.81 (dd, J = 14.5 Hz, 7.5 Hz, 4 H), 4.06 (t, J = 7.0 Hz, 2 H), 6.98 (m, 5 H), 7.07–7.14 (m, 8 H), 7.17 (d, J = 8.5 Hz, 2 H), 7.23–7.25 (m, 3 H), 7.26–7.30 (m, 4 H), 7.46 (d, J = 1.0 Hz, 1 H), 7.49–7.52 (m, 2 H), 7.82 (d, J = 8.5 Hz, 1 H), 7.87 (d, J = 8.5 Hz, 1 H); ^13^C NMR (125.77 MHz, CDCl_3_) δ 151.32, 151.25, 148.45, 148.11, 147.02, 146.82, 146.58, 141.76, 141.71, 136.69, 136.66, 129.16, 123.87, 122.38, 122.06, 121.98, 120.83, 120.76, 119.80, 119.58, 119.45, 118.27, 117.74, 116.91, 111.58, 104.15, 56.09, 42.82, 32.59, 30.95, 20.45, 13.81, 8.63. HRMS calcd. for C_51_H_46_BrN_3_ [*M* + H] m/*z* 780.2948 found 780.2945.

### Refinement

Carbon-bound H-atoms were placed in calculated positions [C—H 0.95 to 0.99 Å, *U*_iso_(H) 1.2 to 1.5*U*_eq_(C)] and were included in the refinement in the riding model approximation. The terminal propyl group of the butyl substituent was found to be disordered. Two sites were resolved and from fractional refinement (common anisotropic displacement parameters for pairs of atoms, and with 1,2- and 1,3- C—C distance constraints = 1.50±0.01 and 2.35±0.01 Å, respectively). The major component had a site occupancy factor = 0.686 (3).

### Crystal data

**Table:**

C_51_H_46_BrN_3_	*F*(000) = 1632
*M**_r_* = 780.82	*D*_x_ = 1.286 Mg m^−^^3^
Monoclinic, *P*2_1_/*n*	Cu *K*α radiation, λ = 1.54184 Å
Hall symbol: -P 2yn	Cell parameters from 7520 reflections
*a* = 8.6585 (2) Å	θ = 3.0–76.8°
*b* = 10.6744 (2) Å	µ = 1.68 mm^−^^1^
*c* = 43.6607 (6) Å	*T* = 100 K
β = 92.114 (2)°	Wedge, colourless
*V* = 4032.56 (13) Å^3^	0.22 × 0.18 × 0.14 mm
*Z* = 4	

### Data collection

**Table:**

Agilent SuperNova Dual diffractometer with an Atlas detector	8220 independent reflections
Radiation source: SuperNova (Cu) X-ray Source	7219 reflections with *I* > 2σ(*I*)
Mirror monochromator	*R*_int_ = 0.025
Detector resolution: 10.4041 pixels mm^-1^	θ_max_ = 75.0°, θ_min_ = 4.1°
ω scan	*h* = −10→10
Absorption correction: multi-scan (*CrysAlis PRO*; Agilent, 2010)	*k* = −13→13
*T*_min_ = 0.836, *T*_max_ = 1.000	*l* = −54→23
17423 measured reflections	

### Refinement

**Table:**

Refinement on *F*^2^	Primary atom site location: structure-invariant direct methods
Least-squares matrix: full	Secondary atom site location: difference Fourier map
*R*[*F*^2^ > 2σ(*F*^2^)] = 0.044	Hydrogen site location: inferred from neighbouring sites
*wR*(*F*^2^) = 0.112	H-atom parameters constrained
*S* = 1.05	*w* = 1/[σ^2^(*F*_o_^2^) + (0.0457*P*)^2^ + 2.7341*P*] where *P* = (*F*_o_^2^ + 2*F*_c_^2^)/3
8220 reflections	(Δ/σ)_max_ < 0.001
506 parameters	Δρ_max_ = 0.39 e Å^−^^3^
28 restraints	Δρ_min_ = −0.66 e Å^−^^3^

### Special details

**Table:**

Geometry. All s.u.'s (except the s.u. in the dihedral angle between two l.s. planes) are estimated using the full covariance matrix. The cell s.u.'s are taken into account individually in the estimation of s.u.'s in distances, angles and torsion angles; correlations between s.u.'s in cell parameters are only used when they are defined by crystal symmetry. An approximate (isotropic) treatment of cell s.u.'s is used for estimating s.u.'s involving l.s. planes.
Refinement. Refinement of *F*^2^ against ALL reflections. The weighted *R*-factor *wR* and goodness of fit *S* are based on *F*^2^, conventional *R*-factors *R* are based on *F*, with *F* set to zero for negative *F*^2^. The threshold expression of *F*^2^ > 2σ(*F*^2^) is used only for calculating *R*-factors(gt) *etc*. and is not relevant to the choice of reflections for refinement. *R*-factors based on *F*^2^ are statistically about twice as large as those based on *F*, and *R*- factors based on ALL data will be even larger.

### Fractional atomic coordinates and isotropic or equivalent isotropic displacement parameters (Å^2^)

**Table:**

	*x*	*y*	*z*	*U*_iso_*/*U*_eq_	Occ. (<1)
Br1	0.49451 (3)	0.07445 (3)	0.162449 (5)	0.04399 (10)	
N1	−0.0180 (3)	0.1837 (2)	0.52239 (4)	0.0408 (5)	
N2	0.4685 (3)	0.59397 (17)	0.35350 (4)	0.0359 (4)	
N3	0.3902 (2)	0.40069 (17)	0.25166 (4)	0.0314 (4)	
C1	0.0477 (3)	0.1430 (2)	0.55124 (5)	0.0382 (5)	
C2	−0.0076 (4)	0.1870 (3)	0.57881 (5)	0.0465 (6)	
H2	−0.0917	0.2441	0.5788	0.056*	
C3	0.0613 (4)	0.1465 (3)	0.60610 (6)	0.0568 (8)	
H3	0.0246	0.1767	0.6250	0.068*	
C4	0.1818 (4)	0.0636 (3)	0.60631 (6)	0.0605 (8)	
H4	0.2292	0.0372	0.6252	0.073*	
C5	0.2344 (4)	0.0182 (3)	0.57888 (7)	0.0578 (8)	
H5	0.3166	−0.0408	0.5789	0.069*	
C6	0.1670 (3)	0.0588 (3)	0.55128 (6)	0.0463 (6)	
H6	0.2036	0.0281	0.5324	0.056*	
C7	−0.1775 (3)	0.1817 (2)	0.51665 (5)	0.0387 (5)	
C8	−0.2718 (3)	0.1027 (2)	0.53283 (6)	0.0434 (6)	
H8	−0.2275	0.0493	0.5482	0.052*	
C9	−0.4296 (3)	0.1006 (2)	0.52690 (6)	0.0464 (6)	
H9	−0.4924	0.0474	0.5387	0.056*	
C10	−0.4982 (3)	0.1748 (2)	0.50412 (6)	0.0442 (6)	
H10	−0.6064	0.1716	0.4998	0.053*	
C11	−0.4046 (3)	0.2534 (2)	0.48794 (5)	0.0424 (6)	
H11	−0.4493	0.3051	0.4723	0.051*	
C12	−0.2482 (3)	0.2583 (2)	0.49409 (5)	0.0405 (6)	
H12	−0.1867	0.3146	0.4829	0.049*	
C13	0.0785 (3)	0.2533 (2)	0.50293 (5)	0.0377 (5)	
C14	0.1720 (3)	0.3484 (2)	0.51471 (5)	0.0402 (6)	
H14	0.1775	0.3625	0.5362	0.048*	
C15	0.2576 (3)	0.4233 (2)	0.49566 (5)	0.0388 (5)	
H15	0.3235	0.4869	0.5040	0.047*	
C16	0.2453 (3)	0.4037 (2)	0.46407 (5)	0.0359 (5)	
C17	0.1538 (3)	0.3060 (2)	0.45225 (5)	0.0344 (5)	
C18	0.0723 (3)	0.2289 (2)	0.47129 (5)	0.0376 (5)	
H18	0.0133	0.1607	0.4632	0.045*	
C19	0.3147 (3)	0.4699 (2)	0.43853 (5)	0.0340 (5)	
C20	0.4145 (3)	0.5718 (2)	0.43754 (5)	0.0366 (5)	
H20	0.4473	0.6131	0.4559	0.044*	
C21	0.4658 (3)	0.6129 (2)	0.40948 (5)	0.0365 (5)	
H21	0.5358	0.6814	0.4088	0.044*	
C22	0.4151 (3)	0.5542 (2)	0.38222 (5)	0.0338 (5)	
C23	0.3134 (3)	0.4527 (2)	0.38326 (5)	0.0336 (5)	
H23	0.2789	0.4124	0.3648	0.040*	
C24	0.2632 (3)	0.4112 (2)	0.41116 (5)	0.0331 (5)	
C25	0.1528 (3)	0.3044 (2)	0.41730 (5)	0.0342 (5)	
C26	−0.0112 (3)	0.3300 (2)	0.40369 (5)	0.0387 (5)	
H26A	−0.0062	0.3341	0.3811	0.046*	
H26B	−0.0784	0.2584	0.4087	0.046*	
C27	−0.0858 (3)	0.4496 (3)	0.41489 (6)	0.0447 (6)	
H27A	−0.1898	0.4577	0.4055	0.067*	
H27B	−0.0231	0.5219	0.4092	0.067*	
H27C	−0.0926	0.4465	0.4372	0.067*	
C28	0.2109 (3)	0.1793 (2)	0.40461 (5)	0.0380 (5)	
H28A	0.1418	0.1114	0.4112	0.046*	
H28B	0.2039	0.1825	0.3819	0.046*	
C29	0.3755 (3)	0.1469 (2)	0.41470 (5)	0.0437 (6)	
H29A	0.4037	0.0658	0.4060	0.066*	
H29B	0.3836	0.1422	0.4371	0.066*	
H29C	0.4457	0.2118	0.4075	0.066*	
C30	0.4771 (3)	0.7215 (2)	0.34542 (5)	0.0335 (5)	
C31	0.3800 (3)	0.8101 (2)	0.35774 (5)	0.0412 (6)	
H31	0.3056	0.7857	0.3720	0.049*	
C32	0.3923 (4)	0.9345 (2)	0.34904 (6)	0.0542 (8)	
H32	0.3260	0.9951	0.3576	0.065*	
C33	0.4991 (4)	0.9723 (3)	0.32808 (7)	0.0582 (9)	
H33	0.5066	1.0579	0.3223	0.070*	
C34	0.5945 (4)	0.8839 (3)	0.31569 (6)	0.0533 (7)	
H34	0.6673	0.9084	0.3011	0.064*	
C35	0.5848 (3)	0.7599 (2)	0.32438 (6)	0.0422 (6)	
H35	0.6523	0.7000	0.3159	0.051*	
C36	0.4962 (3)	0.5007 (2)	0.33102 (5)	0.0320 (5)	
C37	0.6019 (3)	0.4045 (2)	0.33790 (5)	0.0339 (5)	
H37	0.6517	0.4012	0.3576	0.041*	
C38	0.6348 (3)	0.3145 (2)	0.31642 (5)	0.0317 (5)	
H38	0.7063	0.2493	0.3213	0.038*	
C39	0.5614 (3)	0.3203 (2)	0.28737 (5)	0.0286 (4)	
C40	0.4525 (3)	0.4151 (2)	0.28119 (5)	0.0303 (4)	
C41	0.4176 (3)	0.5065 (2)	0.30272 (5)	0.0319 (5)	
H41	0.3433	0.5700	0.2982	0.038*	
C42	0.5683 (3)	0.2456 (2)	0.25990 (5)	0.0290 (4)	
C43	0.6524 (3)	0.1410 (2)	0.25159 (5)	0.0327 (5)	
H43	0.7252	0.1047	0.2658	0.039*	
C44	0.6302 (3)	0.0897 (2)	0.22258 (5)	0.0349 (5)	
H44	0.6873	0.0183	0.2167	0.042*	
C45	0.5223 (3)	0.1448 (2)	0.20215 (5)	0.0333 (5)	
C46	0.4356 (3)	0.2488 (2)	0.20915 (5)	0.0324 (5)	
H46	0.3630	0.2844	0.1948	0.039*	
C47	0.4605 (3)	0.2990 (2)	0.23854 (5)	0.0296 (4)	
C48	0.2643 (3)	0.4762 (2)	0.23805 (5)	0.0357 (5)	
H48A	0.1843	0.4877	0.2534	0.043*	0.314 (3)
H48B	0.2164	0.4296	0.2205	0.043*	0.314 (3)
H48C	0.2073	0.5153	0.2548	0.043*	0.686 (3)
H48D	0.1916	0.4196	0.2268	0.043*	0.686 (3)
C49	0.3146 (5)	0.6037 (3)	0.22698 (8)	0.0396 (9)	0.686 (3)
H49A	0.2215	0.6529	0.2209	0.048*	0.686 (3)
H49B	0.3682	0.6482	0.2442	0.048*	0.686 (3)
C50	0.4191 (4)	0.5989 (3)	0.20061 (8)	0.0440 (8)	0.686 (3)
H50A	0.3715	0.5467	0.1841	0.053*	0.686 (3)
H50B	0.5185	0.5598	0.2072	0.053*	0.686 (3)
C51	0.4481 (6)	0.7292 (4)	0.18859 (11)	0.0651 (12)	0.686 (3)
H51A	0.5068	0.7238	0.1699	0.098*	0.686 (3)
H51B	0.5073	0.7774	0.2041	0.098*	0.686 (3)
H51C	0.3491	0.7709	0.1840	0.098*	0.686 (3)
C49'	0.3129 (14)	0.5782 (7)	0.21644 (15)	0.0396 (9)	0.314 (3)
H49C	0.3716	0.5411	0.1996	0.048*	0.314 (3)
H49D	0.2203	0.6201	0.2073	0.048*	0.314 (3)
C50'	0.4112 (10)	0.6717 (6)	0.23341 (16)	0.0440 (8)	0.314 (3)
H50C	0.5041	0.6302	0.2426	0.053*	0.314 (3)
H50D	0.3527	0.7095	0.2501	0.053*	0.314 (3)
C51'	0.4580 (12)	0.7712 (9)	0.2113 (2)	0.0651 (12)	0.314 (3)
H51D	0.5479	0.7425	0.2003	0.098*	0.314 (3)
H51E	0.4846	0.8480	0.2227	0.098*	0.314 (3)
H51F	0.3722	0.7881	0.1967	0.098*	0.314 (3)

### Atomic displacement parameters (Å^2^)

**Table:**

	*U*^11^	*U*^22^	*U*^33^	*U*^12^	*U*^13^	*U*^23^
Br1	0.06625 (19)	0.03919 (15)	0.02643 (13)	0.00117 (12)	0.00057 (11)	−0.01037 (10)
N1	0.0573 (13)	0.0421 (11)	0.0232 (9)	0.0003 (10)	0.0030 (8)	0.0076 (8)
N2	0.0606 (13)	0.0275 (9)	0.0198 (8)	−0.0020 (9)	0.0055 (8)	−0.0026 (7)
N3	0.0445 (11)	0.0285 (9)	0.0210 (8)	0.0051 (8)	−0.0021 (7)	−0.0025 (7)
C1	0.0570 (15)	0.0344 (12)	0.0233 (10)	−0.0016 (11)	0.0011 (10)	0.0044 (9)
C2	0.0700 (18)	0.0409 (14)	0.0289 (12)	0.0007 (13)	0.0047 (11)	−0.0014 (10)
C3	0.085 (2)	0.0601 (18)	0.0251 (12)	−0.0110 (17)	−0.0002 (13)	−0.0028 (12)
C4	0.070 (2)	0.077 (2)	0.0333 (14)	−0.0104 (17)	−0.0117 (13)	0.0186 (14)
C5	0.0559 (18)	0.0627 (19)	0.0546 (17)	0.0045 (15)	0.0016 (13)	0.0244 (15)
C6	0.0633 (17)	0.0413 (14)	0.0348 (13)	0.0030 (12)	0.0070 (11)	0.0087 (11)
C7	0.0594 (16)	0.0329 (12)	0.0236 (10)	0.0002 (11)	0.0009 (10)	−0.0001 (9)
C8	0.0607 (16)	0.0392 (13)	0.0301 (12)	−0.0017 (12)	−0.0020 (11)	0.0076 (10)
C9	0.0627 (17)	0.0377 (13)	0.0387 (13)	−0.0076 (12)	−0.0006 (12)	0.0059 (11)
C10	0.0542 (15)	0.0390 (13)	0.0388 (13)	−0.0032 (12)	−0.0057 (11)	0.0003 (11)
C11	0.0643 (17)	0.0335 (12)	0.0291 (11)	0.0027 (11)	−0.0023 (11)	0.0013 (10)
C12	0.0603 (16)	0.0341 (12)	0.0272 (11)	0.0014 (11)	0.0045 (10)	0.0025 (9)
C13	0.0556 (15)	0.0337 (12)	0.0241 (10)	0.0020 (11)	0.0031 (10)	0.0037 (9)
C14	0.0666 (17)	0.0349 (12)	0.0192 (10)	0.0017 (11)	0.0010 (10)	0.0007 (9)
C15	0.0651 (16)	0.0307 (11)	0.0206 (10)	−0.0011 (11)	−0.0007 (10)	−0.0007 (9)
C16	0.0582 (15)	0.0293 (11)	0.0200 (10)	0.0024 (10)	0.0005 (9)	0.0008 (8)
C17	0.0522 (14)	0.0300 (11)	0.0209 (10)	0.0015 (10)	−0.0002 (9)	0.0001 (8)
C18	0.0567 (15)	0.0321 (11)	0.0240 (10)	0.0001 (11)	0.0012 (10)	0.0015 (9)
C19	0.0539 (14)	0.0304 (11)	0.0175 (9)	0.0010 (10)	−0.0016 (9)	−0.0011 (8)
C20	0.0575 (15)	0.0328 (11)	0.0192 (10)	−0.0004 (10)	−0.0048 (9)	−0.0042 (8)
C21	0.0541 (15)	0.0317 (11)	0.0236 (10)	−0.0035 (10)	−0.0019 (9)	−0.0016 (9)
C22	0.0527 (14)	0.0306 (11)	0.0181 (9)	0.0021 (10)	0.0006 (9)	−0.0004 (8)
C23	0.0522 (14)	0.0305 (11)	0.0178 (9)	0.0006 (10)	−0.0018 (9)	−0.0018 (8)
C24	0.0505 (13)	0.0280 (10)	0.0205 (10)	0.0019 (10)	−0.0025 (9)	−0.0011 (8)
C25	0.0535 (14)	0.0308 (11)	0.0182 (9)	−0.0005 (10)	−0.0002 (9)	0.0007 (8)
C26	0.0551 (15)	0.0364 (12)	0.0245 (10)	−0.0053 (11)	−0.0005 (10)	0.0005 (9)
C27	0.0602 (17)	0.0451 (14)	0.0287 (11)	0.0053 (12)	−0.0006 (11)	0.0005 (10)
C28	0.0615 (16)	0.0302 (11)	0.0222 (10)	−0.0011 (11)	−0.0016 (10)	−0.0017 (9)
C29	0.0675 (17)	0.0350 (12)	0.0281 (11)	0.0063 (12)	−0.0026 (11)	−0.0010 (10)
C30	0.0505 (14)	0.0293 (11)	0.0204 (9)	−0.0023 (10)	−0.0057 (9)	−0.0019 (8)
C31	0.0609 (16)	0.0378 (13)	0.0242 (10)	0.0063 (11)	−0.0082 (10)	−0.0058 (9)
C32	0.090 (2)	0.0337 (13)	0.0366 (13)	0.0144 (14)	−0.0268 (14)	−0.0116 (11)
C33	0.096 (2)	0.0302 (13)	0.0458 (15)	−0.0121 (14)	−0.0347 (16)	0.0079 (12)
C34	0.073 (2)	0.0442 (15)	0.0415 (14)	−0.0211 (14)	−0.0112 (13)	0.0096 (12)
C35	0.0546 (15)	0.0377 (13)	0.0339 (12)	−0.0064 (11)	−0.0023 (11)	0.0001 (10)
C36	0.0491 (13)	0.0268 (10)	0.0204 (9)	−0.0036 (9)	0.0044 (9)	−0.0012 (8)
C37	0.0488 (13)	0.0319 (11)	0.0208 (9)	−0.0027 (10)	−0.0016 (9)	0.0012 (8)
C38	0.0407 (12)	0.0289 (11)	0.0255 (10)	−0.0005 (9)	−0.0002 (9)	0.0024 (8)
C39	0.0377 (11)	0.0250 (10)	0.0232 (10)	−0.0021 (8)	0.0017 (8)	−0.0001 (8)
C40	0.0427 (12)	0.0274 (10)	0.0208 (9)	−0.0010 (9)	−0.0001 (8)	−0.0002 (8)
C41	0.0446 (13)	0.0280 (10)	0.0232 (10)	0.0029 (9)	0.0019 (9)	−0.0006 (8)
C42	0.0377 (12)	0.0265 (10)	0.0227 (9)	−0.0024 (9)	0.0018 (8)	0.0005 (8)
C43	0.0390 (12)	0.0297 (11)	0.0294 (10)	0.0025 (9)	0.0004 (9)	0.0009 (9)
C44	0.0428 (13)	0.0302 (11)	0.0318 (11)	0.0028 (10)	0.0052 (9)	−0.0035 (9)
C45	0.0442 (13)	0.0316 (11)	0.0241 (10)	−0.0042 (10)	0.0029 (9)	−0.0047 (9)
C46	0.0437 (13)	0.0310 (11)	0.0224 (10)	−0.0012 (9)	−0.0019 (9)	−0.0013 (8)
C47	0.0386 (12)	0.0258 (10)	0.0244 (10)	−0.0011 (9)	0.0017 (8)	−0.0013 (8)
C48	0.0439 (13)	0.0350 (12)	0.0280 (10)	0.0063 (10)	−0.0025 (9)	−0.0009 (9)
C49	0.0516 (17)	0.039 (2)	0.028 (2)	0.0061 (16)	−0.005 (2)	0.0007 (17)
C50	0.0476 (19)	0.0464 (19)	0.0376 (17)	−0.0027 (15)	−0.0033 (14)	0.0048 (15)
C51	0.068 (3)	0.068 (3)	0.059 (2)	−0.008 (2)	−0.002 (2)	0.020 (2)
C49'	0.0516 (17)	0.039 (2)	0.028 (2)	0.0061 (16)	−0.005 (2)	0.0007 (17)
C50'	0.0476 (19)	0.0464 (19)	0.0376 (17)	−0.0027 (15)	−0.0033 (14)	0.0048 (15)
C51'	0.068 (3)	0.068 (3)	0.059 (2)	−0.008 (2)	−0.002 (2)	0.020 (2)

### Geometric parameters (Å, º)

**Table:**

Br1—C45	1.896 (2)	C28—C29	1.516 (4)
N1—C7	1.395 (3)	C28—H28A	0.9900
N1—C13	1.423 (3)	C28—H28B	0.9900
N1—C1	1.430 (3)	C29—H29A	0.9800
N2—C30	1.409 (3)	C29—H29B	0.9800
N2—C22	1.417 (3)	C29—H29C	0.9800
N2—C36	1.425 (3)	C30—C31	1.387 (3)
N3—C47	1.379 (3)	C30—C35	1.394 (4)
N3—C40	1.387 (3)	C31—C32	1.386 (4)
N3—C48	1.464 (3)	C31—H31	0.9500
C1—C6	1.369 (4)	C32—C33	1.385 (5)
C1—C2	1.393 (3)	C32—H32	0.9500
C2—C3	1.382 (4)	C33—C34	1.378 (5)
C2—H2	0.9500	C33—H33	0.9500
C3—C4	1.368 (5)	C34—C35	1.380 (4)
C3—H3	0.9500	C34—H34	0.9500
C4—C5	1.384 (5)	C35—H35	0.9500
C4—H4	0.9500	C36—C41	1.390 (3)
C5—C6	1.389 (4)	C36—C37	1.401 (3)
C5—H5	0.9500	C37—C38	1.380 (3)
C6—H6	0.9500	C37—H37	0.9500
C7—C8	1.386 (4)	C38—C39	1.399 (3)
C7—C12	1.404 (3)	C38—H38	0.9500
C8—C9	1.381 (4)	C39—C40	1.403 (3)
C8—H8	0.9500	C39—C42	1.443 (3)
C9—C10	1.388 (4)	C40—C41	1.395 (3)
C9—H9	0.9500	C41—H41	0.9500
C10—C11	1.379 (4)	C42—C43	1.388 (3)
C10—H10	0.9500	C42—C47	1.415 (3)
C11—C12	1.372 (4)	C43—C44	1.387 (3)
C11—H11	0.9500	C43—H43	0.9500
C12—H12	0.9500	C44—C45	1.397 (3)
C13—C14	1.385 (4)	C44—H44	0.9500
C13—C18	1.405 (3)	C45—C46	1.381 (3)
C14—C15	1.388 (3)	C46—C47	1.400 (3)
C14—H14	0.9500	C46—H46	0.9500
C15—C16	1.395 (3)	C48—C49'	1.511 (5)
C15—H15	0.9500	C48—C49	1.514 (3)
C16—C17	1.397 (3)	C48—H48A	0.9900
C16—C19	1.467 (3)	C48—H48B	0.9900
C17—C18	1.382 (3)	C48—H48C	0.9900
C17—C25	1.526 (3)	C48—H48D	0.9900
C18—H18	0.9500	C49—C50	1.491 (4)
C19—C20	1.391 (3)	C49—H49A	0.9900
C19—C24	1.407 (3)	C49—H49B	0.9900
C20—C21	1.390 (3)	C50—C51	1.511 (4)
C20—H20	0.9500	C50—H50A	0.9900
C21—C22	1.402 (3)	C50—H50B	0.9900
C21—H21	0.9500	C51—H51A	0.9800
C22—C23	1.398 (3)	C51—H51B	0.9800
C23—C24	1.381 (3)	C51—H51C	0.9800
C23—H23	0.9500	C49'—C50'	1.491 (5)
C24—C25	1.518 (3)	C49'—H49C	0.9900
C25—C28	1.538 (3)	C49'—H49D	0.9900
C25—C26	1.543 (3)	C50'—C51'	1.500 (5)
C26—C27	1.520 (4)	C50'—H50C	0.9900
C26—H26A	0.9900	C50'—H50D	0.9900
C26—H26B	0.9900	C51'—H51D	0.9800
C27—H27A	0.9800	C51'—H51E	0.9800
C27—H27B	0.9800	C51'—H51F	0.9800
C27—H27C	0.9800		
			
C7—N1—C13	120.01 (19)	H29B—C29—H29C	109.5
C7—N1—C1	120.8 (2)	C31—C30—C35	119.0 (2)
C13—N1—C1	117.5 (2)	C31—C30—N2	121.6 (2)
C30—N2—C22	122.21 (19)	C35—C30—N2	119.4 (2)
C30—N2—C36	119.42 (18)	C32—C31—C30	119.6 (3)
C22—N2—C36	117.94 (18)	C32—C31—H31	120.2
C47—N3—C40	108.14 (18)	C30—C31—H31	120.2
C47—N3—C48	126.71 (18)	C33—C32—C31	121.3 (3)
C40—N3—C48	125.02 (18)	C33—C32—H32	119.3
C6—C1—C2	120.2 (2)	C31—C32—H32	119.3
C6—C1—N1	118.4 (2)	C34—C33—C32	119.0 (3)
C2—C1—N1	121.3 (2)	C34—C33—H33	120.5
C3—C2—C1	119.2 (3)	C32—C33—H33	120.5
C3—C2—H2	120.4	C33—C34—C35	120.4 (3)
C1—C2—H2	120.4	C33—C34—H34	119.8
C4—C3—C2	120.9 (3)	C35—C34—H34	119.8
C4—C3—H3	119.6	C34—C35—C30	120.8 (3)
C2—C3—H3	119.6	C34—C35—H35	119.6
C3—C4—C5	119.7 (3)	C30—C35—H35	119.6
C3—C4—H4	120.1	C41—C36—C37	121.2 (2)
C5—C4—H4	120.1	C41—C36—N2	119.5 (2)
C4—C5—C6	120.0 (3)	C37—C36—N2	119.35 (19)
C4—C5—H5	120.0	C38—C37—C36	121.0 (2)
C6—C5—H5	120.0	C38—C37—H37	119.5
C1—C6—C5	119.9 (3)	C36—C37—H37	119.5
C1—C6—H6	120.0	C37—C38—C39	119.1 (2)
C5—C6—H6	120.0	C37—C38—H38	120.4
C8—C7—N1	121.1 (2)	C39—C38—H38	120.4
C8—C7—C12	117.5 (2)	C38—C39—C40	119.1 (2)
N1—C7—C12	121.4 (2)	C38—C39—C42	134.2 (2)
C9—C8—C7	120.8 (2)	C40—C39—C42	106.67 (18)
C9—C8—H8	119.6	N3—C40—C41	128.1 (2)
C7—C8—H8	119.6	N3—C40—C39	109.50 (18)
C8—C9—C10	121.3 (3)	C41—C40—C39	122.36 (19)
C8—C9—H9	119.3	C36—C41—C40	117.2 (2)
C10—C9—H9	119.3	C36—C41—H41	121.4
C11—C10—C9	118.1 (3)	C40—C41—H41	121.4
C11—C10—H10	121.0	C43—C42—C47	119.45 (19)
C9—C10—H10	121.0	C43—C42—C39	134.4 (2)
C12—C11—C10	121.1 (2)	C47—C42—C39	106.18 (19)
C12—C11—H11	119.4	C44—C43—C42	120.0 (2)
C10—C11—H11	119.4	C44—C43—H43	120.0
C11—C12—C7	121.1 (2)	C42—C43—H43	120.0
C11—C12—H12	119.4	C43—C44—C45	118.9 (2)
C7—C12—H12	119.4	C43—C44—H44	120.5
C14—C13—C18	120.1 (2)	C45—C44—H44	120.5
C14—C13—N1	120.6 (2)	C46—C45—C44	123.6 (2)
C18—C13—N1	119.2 (2)	C46—C45—Br1	117.97 (17)
C13—C14—C15	121.1 (2)	C44—C45—Br1	118.43 (17)
C13—C14—H14	119.4	C45—C46—C47	116.3 (2)
C15—C14—H14	119.4	C45—C46—H46	121.8
C14—C15—C16	118.9 (2)	C47—C46—H46	121.8
C14—C15—H15	120.6	N3—C47—C46	128.8 (2)
C16—C15—H15	120.6	N3—C47—C42	109.50 (18)
C15—C16—C17	119.9 (2)	C46—C47—C42	121.7 (2)
C15—C16—C19	131.3 (2)	N3—C48—C49'	115.4 (5)
C17—C16—C19	108.72 (19)	N3—C48—C49	113.9 (3)
C18—C17—C16	121.2 (2)	N3—C48—H48A	108.8
C18—C17—C25	127.6 (2)	C49'—C48—H48A	123.5
C16—C17—C25	111.1 (2)	C49—C48—H48A	108.8
C17—C18—C13	118.6 (2)	N3—C48—H48B	108.8
C17—C18—H18	120.7	C49'—C48—H48B	89.9
C13—C18—H18	120.7	C49—C48—H48B	108.8
C20—C19—C24	120.0 (2)	H48A—C48—H48B	107.7
C20—C19—C16	132.3 (2)	N3—C48—H48C	108.4
C24—C19—C16	107.8 (2)	C49'—C48—H48C	108.4
C21—C20—C19	119.6 (2)	C49—C48—H48C	90.8
C21—C20—H20	120.2	H48B—C48—H48C	125.2
C19—C20—H20	120.2	N3—C48—H48D	108.4
C20—C21—C22	120.5 (2)	C49'—C48—H48D	108.4
C20—C21—H21	119.8	C49—C48—H48D	125.1
C22—C21—H21	119.8	H48A—C48—H48D	88.0
C23—C22—C21	119.8 (2)	H48C—C48—H48D	107.5
C23—C22—N2	119.17 (19)	C50—C49—C48	113.9 (3)
C21—C22—N2	121.0 (2)	C50—C49—H49A	108.8
C24—C23—C22	119.7 (2)	C48—C49—H49A	108.8
C24—C23—H23	120.1	C50—C49—H49B	108.8
C22—C23—H23	120.1	C48—C49—H49B	108.8
C23—C24—C19	120.4 (2)	H49A—C49—H49B	107.7
C23—C24—C25	128.1 (2)	C49—C50—C51	110.5 (3)
C19—C24—C25	111.48 (19)	C49—C50—H50A	109.6
C24—C25—C17	100.81 (18)	C51—C50—H50A	109.6
C24—C25—C28	111.8 (2)	C49—C50—H50B	109.6
C17—C25—C28	112.35 (18)	C51—C50—H50B	109.6
C24—C25—C26	112.07 (19)	H50A—C50—H50B	108.1
C17—C25—C26	110.7 (2)	C50—C51—H51A	109.5
C28—C25—C26	108.90 (19)	C50—C51—H51B	109.5
C27—C26—C25	114.9 (2)	H51A—C51—H51B	109.5
C27—C26—H26A	108.5	C50—C51—H51C	109.5
C25—C26—H26A	108.5	H51A—C51—H51C	109.5
C27—C26—H26B	108.5	H51B—C51—H51C	109.5
C25—C26—H26B	108.5	C50'—C49'—C48	109.8 (5)
H26A—C26—H26B	107.5	C50'—C49'—H49C	109.7
C26—C27—H27A	109.5	C48—C49'—H49C	109.7
C26—C27—H27B	109.5	C50'—C49'—H49D	109.7
H27A—C27—H27B	109.5	C48—C49'—H49D	109.7
C26—C27—H27C	109.5	H49C—C49'—H49D	108.2
H27A—C27—H27C	109.5	C49'—C50'—C51'	108.5 (5)
H27B—C27—H27C	109.5	C49'—C50'—H50C	110.0
C29—C28—C25	114.20 (19)	C51'—C50'—H50C	110.0
C29—C28—H28A	108.7	C49'—C50'—H50D	110.0
C25—C28—H28A	108.7	C51'—C50'—H50D	110.0
C29—C28—H28B	108.7	H50C—C50'—H50D	108.4
C25—C28—H28B	108.7	C50'—C51'—H51D	109.5
H28A—C28—H28B	107.6	C50'—C51'—H51E	109.5
C28—C29—H29A	109.5	H51D—C51'—H51E	109.5
C28—C29—H29B	109.5	C50'—C51'—H51F	109.5
H29A—C29—H29B	109.5	H51D—C51'—H51F	109.5
C28—C29—H29C	109.5	H51E—C51'—H51F	109.5
H29A—C29—H29C	109.5		
			
C7—N1—C1—C6	130.2 (3)	C18—C17—C25—C26	60.7 (3)
C13—N1—C1—C6	−64.6 (3)	C16—C17—C25—C26	−116.1 (2)
C7—N1—C1—C2	−49.5 (4)	C24—C25—C26—C27	−57.3 (3)
C13—N1—C1—C2	115.7 (3)	C17—C25—C26—C27	54.4 (3)
C6—C1—C2—C3	1.3 (4)	C28—C25—C26—C27	178.4 (2)
N1—C1—C2—C3	−178.9 (3)	C24—C25—C28—C29	52.0 (3)
C1—C2—C3—C4	−0.5 (5)	C17—C25—C28—C29	−60.6 (3)
C2—C3—C4—C5	−0.8 (5)	C26—C25—C28—C29	176.4 (2)
C3—C4—C5—C6	1.3 (5)	C22—N2—C30—C31	−27.5 (3)
C2—C1—C6—C5	−0.8 (4)	C36—N2—C30—C31	144.8 (2)
N1—C1—C6—C5	179.4 (3)	C22—N2—C30—C35	153.0 (2)
C4—C5—C6—C1	−0.5 (5)	C36—N2—C30—C35	−34.7 (3)
C13—N1—C7—C8	171.9 (2)	C35—C30—C31—C32	−0.3 (3)
C1—N1—C7—C8	−23.3 (4)	N2—C30—C31—C32	−179.8 (2)
C13—N1—C7—C12	−7.4 (3)	C30—C31—C32—C33	0.4 (4)
C1—N1—C7—C12	157.5 (2)	C31—C32—C33—C34	0.2 (4)
N1—C7—C8—C9	−179.6 (2)	C32—C33—C34—C35	−0.9 (4)
C12—C7—C8—C9	−0.3 (4)	C33—C34—C35—C30	1.0 (4)
C7—C8—C9—C10	1.7 (4)	C31—C30—C35—C34	−0.5 (4)
C8—C9—C10—C11	−1.6 (4)	N2—C30—C35—C34	179.1 (2)
C9—C10—C11—C12	0.1 (4)	C30—N2—C36—C41	−50.9 (3)
C10—C11—C12—C7	1.3 (4)	C22—N2—C36—C41	121.7 (2)
C8—C7—C12—C11	−1.2 (4)	C30—N2—C36—C37	129.3 (2)
N1—C7—C12—C11	178.1 (2)	C22—N2—C36—C37	−58.1 (3)
C7—N1—C13—C14	121.1 (3)	C41—C36—C37—C38	2.0 (4)
C1—N1—C13—C14	−44.3 (3)	N2—C36—C37—C38	−178.2 (2)
C7—N1—C13—C18	−55.7 (3)	C36—C37—C38—C39	0.3 (3)
C1—N1—C13—C18	139.0 (2)	C37—C38—C39—C40	−2.1 (3)
C18—C13—C14—C15	1.8 (4)	C37—C38—C39—C42	179.2 (2)
N1—C13—C14—C15	−175.0 (2)	C47—N3—C40—C41	179.1 (2)
C13—C14—C15—C16	1.7 (4)	C48—N3—C40—C41	−4.8 (4)
C14—C15—C16—C17	−3.0 (4)	C47—N3—C40—C39	−1.2 (3)
C14—C15—C16—C19	177.0 (3)	C48—N3—C40—C39	174.9 (2)
C15—C16—C17—C18	1.0 (4)	C38—C39—C40—N3	−177.8 (2)
C19—C16—C17—C18	−179.0 (2)	C42—C39—C40—N3	1.2 (2)
C15—C16—C17—C25	178.1 (2)	C38—C39—C40—C41	1.9 (3)
C19—C16—C17—C25	−1.9 (3)	C42—C39—C40—C41	−179.1 (2)
C16—C17—C18—C13	2.4 (4)	C37—C36—C41—C40	−2.1 (3)
C25—C17—C18—C13	−174.2 (2)	N2—C36—C41—C40	178.1 (2)
C14—C13—C18—C17	−3.7 (4)	N3—C40—C41—C36	179.8 (2)
N1—C13—C18—C17	173.1 (2)	C39—C40—C41—C36	0.2 (3)
C15—C16—C19—C20	0.4 (5)	C38—C39—C42—C43	−1.6 (5)
C17—C16—C19—C20	−179.5 (3)	C40—C39—C42—C43	179.7 (2)
C15—C16—C19—C24	−179.8 (3)	C38—C39—C42—C47	178.0 (2)
C17—C16—C19—C24	0.2 (3)	C40—C39—C42—C47	−0.7 (2)
C24—C19—C20—C21	−1.7 (4)	C47—C42—C43—C44	−0.1 (3)
C16—C19—C20—C21	178.0 (3)	C39—C42—C43—C44	179.5 (2)
C19—C20—C21—C22	1.4 (4)	C42—C43—C44—C45	−0.1 (3)
C20—C21—C22—C23	−0.6 (4)	C43—C44—C45—C46	0.2 (4)
C20—C21—C22—N2	−179.0 (2)	C43—C44—C45—Br1	179.46 (18)
C30—N2—C22—C23	136.5 (2)	C44—C45—C46—C47	0.0 (4)
C36—N2—C22—C23	−35.9 (3)	Br1—C45—C46—C47	−179.32 (17)
C30—N2—C22—C21	−45.1 (4)	C40—N3—C47—C46	−179.9 (2)
C36—N2—C22—C21	142.5 (2)	C48—N3—C47—C46	4.0 (4)
C21—C22—C23—C24	0.1 (4)	C40—N3—C47—C42	0.7 (3)
N2—C22—C23—C24	178.6 (2)	C48—N3—C47—C42	−175.3 (2)
C22—C23—C24—C19	−0.4 (4)	C45—C46—C47—N3	−179.5 (2)
C22—C23—C24—C25	179.4 (2)	C45—C46—C47—C42	−0.2 (3)
C20—C19—C24—C23	1.2 (4)	C43—C42—C47—N3	179.7 (2)
C16—C19—C24—C23	−178.6 (2)	C39—C42—C47—N3	0.0 (2)
C20—C19—C24—C25	−178.7 (2)	C43—C42—C47—C46	0.3 (3)
C16—C19—C24—C25	1.6 (3)	C39—C42—C47—C46	−179.4 (2)
C23—C24—C25—C17	177.6 (2)	C47—N3—C48—C49'	−83.2 (4)
C19—C24—C25—C17	−2.5 (3)	C40—N3—C48—C49'	101.4 (4)
C23—C24—C25—C28	58.1 (3)	C47—N3—C48—C49	−105.5 (3)
C19—C24—C25—C28	−122.1 (2)	C40—N3—C48—C49	79.0 (3)
C23—C24—C25—C26	−64.5 (3)	N3—C48—C49—C50	66.4 (4)
C19—C24—C25—C26	115.3 (2)	C49'—C48—C49—C50	−32.5 (15)
C18—C17—C25—C24	179.5 (2)	C48—C49—C50—C51	173.2 (3)
C16—C17—C25—C24	2.6 (3)	N3—C48—C49'—C50'	−63.3 (9)
C18—C17—C25—C28	−61.3 (3)	C49—C48—C49'—C50'	27.2 (11)
C16—C17—C25—C28	121.8 (2)	C48—C49'—C50'—C51'	179.8 (8)

### Hydrogen-bond geometry (Å, º)

Cg1–Cg3 are the centroids of the C13–C18, C7–C12 and C36···C41 rings, respectively.

**Table:**

*D*—H···*A*	*D*—H	H···*A*	*D*···*A*	*D*—H···*A*
C10—H10···*Cg*1^i^	0.95	2.67	3.444 (3)	139
C20—H20···*Cg*2^ii^	0.95	2.85	3.579 (2)	135
C44—H44···*Cg*3^iii^	0.95	2.99	3.836 (3)	149

Symmetry codes: (i) *x*−1, *y*, *z*; (ii) −*x*, −*y*+1, −*z*+1; (iii) *x*+3/2, −*y*−1/2, *z*+1/2.
